# A strategy to enhance the safety and efficiency of handovers of ICU patients: study protocol of the pICUp study

**DOI:** 10.1186/1748-5908-8-67

**Published:** 2013-06-14

**Authors:** Nelleke van Sluisveld, Marieke Zegers, Gert Westert, Johannes Gerardus van der Hoeven, Hub Wollersheim

**Affiliations:** 1 Scientific Institute for Quality of Healthcare (IQ healthcare), Radboud University Nijmegen Medical Centre, PO Box 9101, Nijmegen 6500 HB, The Netherlands; 2 Department of Intensive Care Medicine, Radboud University Nijmegen Medical Centre, PO Box 9101, Nijmegen 6500 HB, the Netherlands; 3 Academic Center for Primary Care, Catholic University Leuven, Kapucijnenvoer, B-3000 Leuven, Belgium

**Keywords:** Intensive care, Critical care, Patient safety, Quality of healthcare, Patient handoff, Patient readmission, Hospital mortality, Guideline adherence, Implementation

## Abstract

**Background:**

To use intensive care unit (ICU) facilities efficiently and ensure high quality of care, an optimal patient flow is necessary. Discharging patients relieves the pressure on ICU beds but the risk of premature discharge must be managed carefully. Suboptimal patient discharge may result in ICU readmissions and in patients’ death.

The aim of this study is to obtain insight into the safety and efficiency of current ICU discharge practices and into barriers and facilitators to the implementation of effective ICU discharge interventions, and to develop an implementation strategy tailored to the barriers and facilitators identified.

**Methods/design:**

This study exists of five phases. Phase A: analysis of routinely registered data on variation in ICU readmissions and hospital mortality after ICU discharge of all ICUs participating in the Dutch National Intensive Care Evaluation registry (n = 83). Phase B: systematic review of effective interventions aiming to improve the efficiency and safety of the ICU discharge process. Phase C: assessing the intervention adherence with a questionnaire survey among all Dutch ICUs (n = 90). Phase D: assessing barriers and facilitators to the implementation of effective ICU discharge interventions with a questionnaire survey among all Dutch intensivists (n = 700). The questionnaire will be based on barriers and facilitators identified by focus groups (n = 4) and individual interviews with professionals of ICUs and general wards and adult discharged ICU patients (n = 25 to 30). Phase E: systematic development of an implementation strategy based on the sampled data in phase A to D, and effective implementation strategies from the literature using the intervention mapping method.

**Discussion:**

Using theory and empirical data, an implementation strategy will be developed to improve the safety and efficiency of the ICU discharge process. The developed strategy will be evaluated in a subsequent study. The knowledge obtained in this study should be used for further implementation of ICU discharge interventions, and can be used for implementation of handover interventions in other healthcare transition settings.

## Background

The intensive care unit (ICU) is an essential component of most large hospitals, providing critically ill patients with high quality care. In addition, patients undergoing major surgery often require ICU admission postoperatively [1]. Therefore, ICUs are often under forward pressure from operating theatres and the emergency room for beds [2]. At the moment, ICU facilities are scarce, and the need for ICU beds will increase in the future as the population ages. Although ICU beds comprise less than 10% of hospital beds, ICU departments consume up to 22% of total hospital costs in the United States [3,4]. As a consequence, efficient use of ICU facilities has become a priority.

An optimal patient flow is critical to use ICU facilities efficiently and to ensure high quality of care. Discharging patients is one way of relieving the pressure on ICU beds but clearly, the risk of premature discharge must be managed carefully [1]. The increased pressure on ICU beds may result in premature and suboptimal discharge leading to ICU readmissions and even in patients’ death [5-7].

ICU readmission rates and hospital mortality after ICU discharge vary. Hospital mortality after ICU discharge is 12.4% in the United Kingdom, of which 39% is related to premature ICU discharge [8]. Other studies show a variation in hospital mortality rates after ICU discharge between 4.5% and 12.4% [8-13]. ICU readmission rates vary between 0.89% and 19% [12,14-16].

ICU readmissions are an important cost driver. The mean unit price of an ICU day in the Netherlands is €2,183 [17]. According to the Dutch National Intensive Care Evaluation (NICE) registry in 2011, approximately 75,000 patients were admitted to the ICU. A reduction of the readmissions rate with 1% (from 6.8% to 5.8%), assuming a median ICU stay of one day, could save 1.6 million euro per year.

The question is: How to prevent ICU readmissions and mortality after an ICU stay? What are available effective improvement interventions and are they used in daily practice? In addition to existing guidelines [18-21], literature describes several evidence-based interventions that focus on organizational changes to improve the safety and efficiency of the ICU discharge process, such as discharge planning [22], monitoring of post-ICU patients [23], medication reconciliation [24], and ICU liaison nurses [25,26]. Adoption of guidelines and improvement interventions in clinical practice has proven to be difficult. Adherence to guidelines and interventions may be hindered by a variety of barriers [27,28]. Better implementation of existing guidelines and interventions aimed at improving the handover of patients from the ICU to general wards may reduce ICU readmissions and hospital mortality. As a positive side effect, avoiding ICU readmissions will reduce hospital costs substantially.

### Objective

The aim of the pICUp (Patient Handover Intensive Care Unit Improvement) study is to obtain insight into current ICU discharge practice, an overview of effective ICU discharge interventions and into the factors that hinder and facilitate the implementation of these interventions (barriers and facilitators), with the final aim to develop a tailored implementation strategy. A better understanding of the barriers and facilitators and a tailored strategy will enhance the implementation of interventions in daily practice and will improve the quality of the ICU discharge process, leading to fewer adverse patient outcomes, such as fewer ICU readmissions and reduced hospital mortality after ICU discharge.

The following research questions are formulated to address this aim:

1. What is the variation in ICU readmissions and hospital mortality after ICU discharge between ICUs?

2. What are effective ICU discharge interventions to prevent ICU readmissions and hospital mortality after ICU discharge?

3. What is the adherence to these interventions?

4. What are the barriers and facilitators to the implementation of these ICU discharge interventions?

5. What is an appropriate strategy to improve the implementation of these ICU discharge interventions?

### Theoretical framework

**Figure 1:**
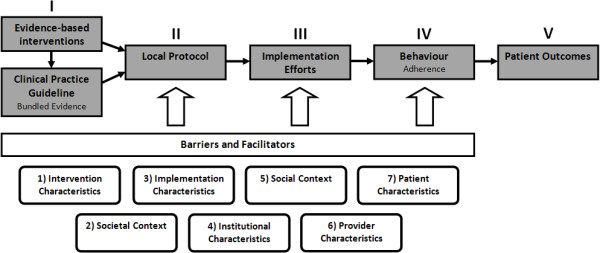
Framework for the implementation of guidelines and interventions.

To answer the research questions and structure the analysis, we developed a framework that provides insight into the process of the implementation of scientific evidence, and factors influencing this process (Figure 1). This framework is based on three models related to implementing change: the implementation of change model of Grol and Wensing [27,29]; the framework of knowledge–attitude–behaviour related barriers for guideline adherence of Cabana *et al.*[28]; and the framework for adherence to clinical practice guidelines in the ICU of Cahill *et al.*[30].

The gray boxes reflect the temporal sequence of the implementation process from scientific evidence to improved patient outcomes: I) scientific research results in evidence about effective interventions that are recommended in clinical practice guidelines, II) the evidence should be tailored to local circumstances in local protocols, III) implementation efforts, such as a detailed and feasible implementation plan, and engaging stakeholders in an early stage, will improve the implementation process, leading to IV) improved behaviour and adherence to the intervention of stakeholders, and ultimately resulting in V) improved patient outcomes.

Several factors influence the implementation process and could explain why effective and evidence-based interventions are not (fully) implemented. Based on literature, barriers and facilitators to implementation can be categorized in seven main domains [27-29,31], represented by the white boxes in Figure 1. The domains are related to the: 1) characteristics of the intervention; 2) societal context; 3) characteristics of the implementation efforts; 4) characteristics of the healthcare facility; 5) social context (*e.g.*, interpersonal, interdepartmental and interinstitutional relationships); 6) professional characteristics; and 7) patient characteristics. Subcategories of these main domains are presented in Table 1.

**Table 1:** **Theoretical framework for classifying barriers and facilitators, based on Grol and Wensing, Cabana *et al. *and van Sluisveld *et al. *[**[27]**-**[29]**,**[31]**]**

**Levels**	**Sublevels**
**Intervention characteristics**	Advantages in Practice, Feasibility, Credibility, Accessibility, Attractiveness, Usefulness, Presence of Contradictory Guidelines
**Societal context**	Social developments, Political developments and policies, Legal obligations and regulations, Financial arrangements, Moral objections
**Implementation characteristics**	Protocol, Implementation strategy, Exposure to implementation efforts
**Institutional characteristics**	Organization of care processes, Organizational structure, Time, Staff, Capacities, Resources, Structures, Technical Support
**Social context**	Culture of social network, Opinion of colleagues, Leadership, Collaboration
**Provider characteristics**	Cognition, Awareness, Attitude, Motivation, Knowledge, Skills, Behavioural Routines
**Patient characteristics**	Compliance, Knowledge, Skills, Attitude, Preferences

Examples of influencing factors related to the intervention are the feasibility to actually incorporate the intervention in daily practice, the credibility of evidence behind the intervention, and the advantages for the healthcare workers or the patients. Societal factors are whether an intervention is reimbursed by healthcare insurers, political climate, policies, and regulations. Factors related to the implementation characteristics are, for example, the availability of an implementation plan, adequate education of professionals, and the degree of exposure of the professionals to the implementation efforts. Influencing institutional factors are for example organizational structure and resource availability. Social interactions within a team or within the network of a healthcare provider may also be of influence; collaboration between care providers or between wards, leadership, and (safety) culture are important factors. Main professional factors are knowledge (such as familiarity with guideline) and attitude (agreement, outcome expectancy, perceived behavioural control) [28]. Patient characteristics, such as compliance, knowledge, and attitude (such as self-efficacy, subjective norms, degree of confidence) may influence the adherence of the healthcare providers to the intervention.

Currently, 30% to 40% of all patients do not receive care according to actual scientific knowledge [32]. We have a limited understanding of the specific factors that determine the success or failure of the implementation of ICU discharge interventions. Identifying these factors may assist in designing tailored and thus more effective implementation strategies [30].

## Study design and methods

### Study design

The pICUp study is a descriptive, explorative study using a mixed method design. Quantitative methods are analysis of registered clinical data and questionnaire surveys, while qualitative methods (individual interviews and focus group interviews) study variation in patient outcomes, current ICU discharge practice, guideline and intervention adherence, and the barriers and facilitators to implementation. Based on these findings a tailor-made implementation strategy will be developed.

**Figure 2:**
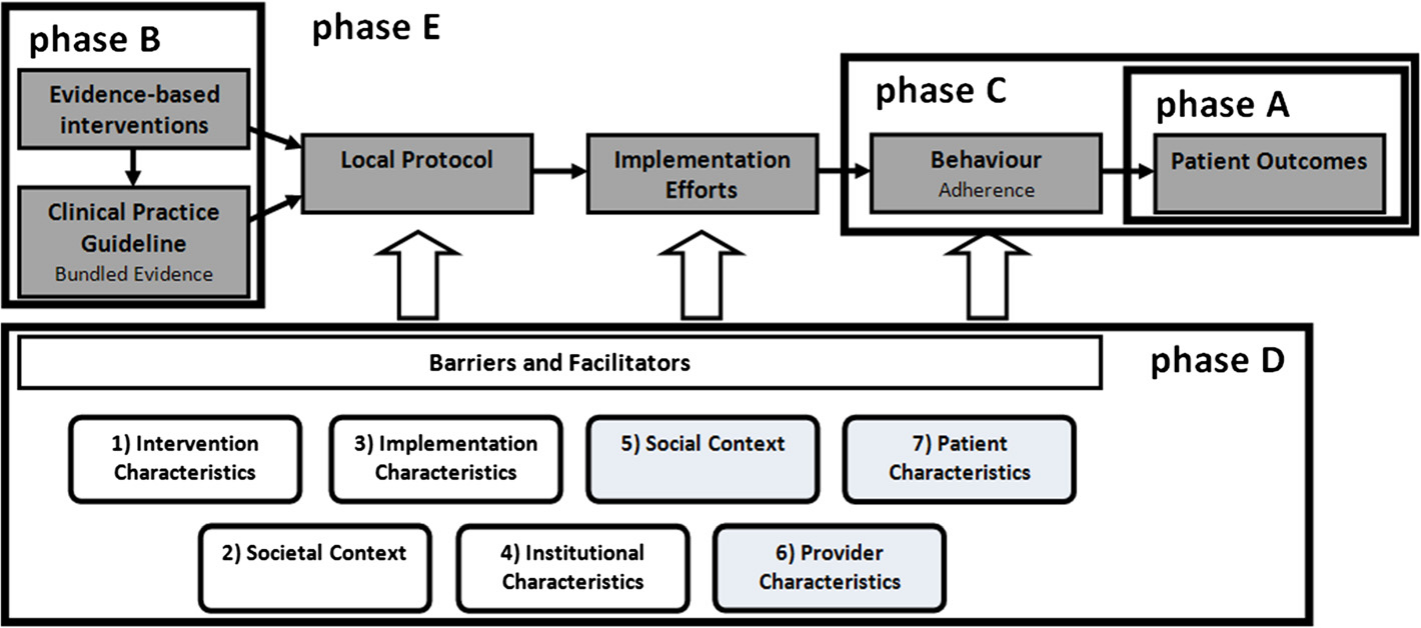
Framework for the implementation of guidelines and interventions including the study phases.

According to the research questions, the study is divided into five phases (Figure 2):

A. Analysis of the variation in ICU readmissions and hospital mortality after ICU discharge between ICUs,

B. Systematic review of evidence-based interventions for improving handover of patients from the ICU to general wards,

C. Analysis of guideline and intervention adherence and the association between adherence and patient outcomes,

D. Analysis of barriers and facilitators to the implementation of ICU discharge guidelines and interventions and the relevance of these factors to professionals,

E. Development of an implementation strategy tailored to the barriers and facilitators found.

Below, we describe the study methods, population, analysis, and outcome measures per study phase. An overview of the research questions, methods and outcomes is given in Table 2.

**Table 2:** Research questions, methods, study population, and outcome measures

**Stage**	**Research questions**	**Methods**	**Target group/data resource**	**Outcome measures**
A	What is the variation in patient outcomes regarding ICU discharges?	Analyzing variation in quality of care	Data from 2011 from adult patients of all Dutch ICUs participating in the NICE registry (n = 84).	ICU quality indicators, *e.g.*:
				- ICU readmissions
				- ICU and hospital mortality
B	What are effective interventions to improve the safety of the ICU discharge process?	Systematic review	Pubmed (including Medline), Cinahl, PsychInfo, Cochrane database, and EMBASE	Overview of effective interventions and effect sizes
C	What is the adherence to guidelines and effective ICU discharge interventions?	Questionnaire to explore guideline adherence and use of effective ICU discharge interventions	All Dutch ICUs (n = 94)	Scale implementation problem: adherence to ICU discharge guidelines and the use of effective interventions. Association between scores on ICU quality indicators (research question 1) and guidelines and intervention adherence (research question 2).
D	What are the barriers and facilitators to the implementation of guidelines and effective ICU discharge interventions?	Individual semi structured (n = 25–30) and focus group interviews (n = 4)	Intensivists, physicians, nurses and managers of ICUs and general wards and patients and their relatives.	Barriers and facilitators classified according to: (1) intervention characteristics; (2) societal context, (3) implementation characteristics, (4) institutional characteristics, (5) social context, (6) professional characteristics, and (7) patient characteristics.
		Questionnaire to quantify the barriers and facilitators from the individual and focus group interviews	All Dutch intensivists (n = 700).	
E	What is an appropriate strategy to implement effective interventions to improve the safety and efficiency of the ICU discharge procedure?	Intervention mapping with the method of Bartholomew and Kok (2011)	Matching the data from the interviews, focus groups and questionnaires to effective implementation strategies from the literature	Implementation strategy tailored to the found barriers and facilitators to the implementation of effective ICU discharge interventions

### Phase A: Analysis of the variation in ICU readmissions and hospital mortality after ICU discharge between ICUs

The aim of this phase is to analyze the variation in ICU readmissions and hospital mortality after ICU discharge between ICUs, and the degree of variation not explained by patient mix.

### Study design and population

Data about the quality indicators related to suboptimal ICU discharge are derived from the Dutch NICE registry. Since 1996, demographic, physiological, and clinical data of patients admitted to Dutch ICUs are collected. All participating ICUs are obliged to attend training in accurate collection of data to ensure the quality of the registry. At each individual ICU and centrally, data are automatically checked for range and consistency. In addition, quality audits are carried out to ensure the validity of the registration [33,34]. Data of 2011 will be used in this study, in which 83 of the in total 90 Dutch ICUs (92%) participated. In that year, the data of over 74,000 patients of 18 years and older have been collected.

### Outcome measures

The variation in patient outcomes will be analyzed using ICU quality indicators related to suboptimal ICU discharge [35,36]. The primary outcome measure is the ICU readmission rate. It is generally assumed that the shorter the time between discharge and readmission, the more likely the ICU discharge was premature. Therefore, readmissions within 48 hours are considered to be related to the quality of the discharge process [15,37,38].

The secondary patient outcome measure is hospital mortality after ICU discharge, which is defined as the percentage of ICU patients discharged alive from the ICU who died on a general ward.

In addition, the association between patient’s outcomes and discharge time will be analyzed, because we know from previous studies that patients discharged at night experience a greater risk of mortality than patients discharged during the day [1,6,39].

### Analysis

Descriptive statistics will be used to characterize the study sample and to report the variation in hospital mortality rates after ICU discharge and ICU readmission rates. To attribute variation to suboptimal care, the rates will be corrected for patient mix (*e.g.*, age, APACHE IV score, co-morbidity at admission, diagnosis at admission, reason for discharge) and organizational factors (*e.g.*, hospital type and ICU level) using multi level analysis. The remaining variance indicates room for improvement [40].

### Phase B: systematic review of evidence-based interventions for improving handover of patients from the ICU to general wards

The aim of this phase is to systematically review literature on effective interventions that aim to improve patient handovers between ICUs and general hospital wards and to evaluate their overall effects.

### Methods

We will search for studies using PubMed (including Medline), Cinahl, PsychInfo, the Cochrane Library, and EMBASE. The inclusion criteria will be: studies with experimental study designs; that include patients undergoing and/or healthcare providers involved in the transition from ICU to ward; that have an intervention explicitly describing one or more components aiming to improve the handover from ICU to ward; and that have ICU readmission rate or hospital mortality rate after ICU admission as an outcome measure.

First, studies that do not meet the inclusion criteria will be eliminated based on their title and/or abstract. Full-text copies of studies identified as potentially relevant will be retrieved and reviewed for the final inclusion. The methodological quality of the included studies will be assessed and data such as a description of objectives, design, participants, intervention, and effect measures will be extracted.

### Analysis

The study outcomes will be presented in tabular form, and a qualitative assessment will be made based on the methodological quality, sample size, intervention characteristics, outcome, statistical significance, and effect size.

### Phase C: analysis of guideline and intervention adherence and the association between adherence and patient outcomes

The aim of this phase is to assess the adherence to the Dutch national guideline [18] and interventions aimed at improving the handover process (such as discharge planning [22], medication reconciliation [24], step down beds [41], monitoring of post-ICU patients [23], and ICU liaison nurses [25,26]), and to analyze the relation between adherence and patient outcomes.

### Study design and population

The intervention adherence will be studied using a questionnaire survey, which includes questions about local policies, organization, and procedures regarding the ICU discharge process and the application of ICU discharge interventions. Questions from the questionnaire of the Dutch Healthcare Inspectorate (IGZ) and the visitation questionnaire of the Dutch Society of Intensive Care (NVIC) will be used [35,36]. Moreover, questions will be formulated about interventions derived from the literature review. The questionnaire will be sent to all ICUs in the Netherlands (n = 90).

The association between intervention adherence (questionnaire) and patient outcomes (Phase B) will be analyzed to determine whether adherence leads to better patient outcomes.

### Analysis

Descriptive statistics will be used to characterize the study sample and to report the adherence to the guideline and interventions. Regression analysis will be performed to analyze the association between adherence and patient outcomes. Adherence to each discharge intervention will be dichotomised, resulting in one adherence score.

### Phase D: analysis of barriers and facilitators to the implementation of ICU discharge guidelines and interventions and the relevance of these factors to professionals

The aim of this phase is to explore the factors influencing the implementation of the ICU discharge guideline [18] and interventions aimed at improving the ICU discharge process [22-26,41].

### Study design

A combination of qualitative and quantitative methods will be used to identify and quantify barriers and facilitators to ICU discharge guideline and intervention adherence.

First, semi-structured interviews will be conducted to explore all relevant barriers and facilitators to guideline and intervention adherence and opportunities for improvement. The interview questions will be based on a loose structure consisting of open-ended questions that define the area to be explored.

Second, focus group interviews will be conducted to gain broader insight into the barriers and facilitators. For both the individual interviews and the focus groups, an interview guideline will be formulated with a series of open-ended questions to explore barriers and facilitators to inappropriate ICU discharge processes and regarding the implementation of the ICU discharge interventions.

Third, a questionnaire will be developed to quantify the barriers and facilitators identified in the individual and focus group interviews. The questionnaire will contain questions about demographic characteristics, and statements concerning barriers and facilitators regarding the implementation of ICU discharge guideline and interventions identified.

### Study population

Approximately 25 to 30 individual interviews will be carried out with managers and healthcare professionals from ICUs and receiving general wards, including intensivists, ICU nurses, and physicians and nurses of general wards. They will be recruited from six hospitals: two general, two teaching, and two academic hospitals.

Furthermore, patients will be interviewed together with a relative, because many post-ICU patients do not remember the ICU admission and the period immediately afterward. The amount of interviews depends on the point of saturation: when no new analytical constructs can be identified in interviews and focus groups [42].

Four focus group interviews (moderated group discussions with six to ten persons) will be performed with intensivists, ICU nurses, physicians of general wards, and nurses of general wards. They will be recruited from several ICUs, ensuring a representative sample in terms of ICU size.

The questionnaire will be sent to all intensivists in the Netherlands registered with the Dutch Society of Intensive Care (NVIC) to quantify barriers and facilitators identified in the individual and focus group interviews.

### Analysis

The individual and focus group interviews will be recorded and transcribed verbatim according to a standardized format. The transcripts will be analyzed and coded by a researcher with qualitative data analysis software (Atlas.ti). The barriers and facilitators will be classified according to analytical categories, based on the framework described in section ‘theoretical framework’.

Descriptive statistics will be used to characterize the study sample and to report the results of the interviews, focus groups, and questionnaires. Variance in outcomes from the questionnaire between different types of hospitals (university and non-university hospitals) and demographic characters (age, gender, and years of experience) will be analyzed.

### Phase E: development of an implementation strategy tailored to the barriers and facilitators found

The aim of this phase is to develop a tailored implementation strategy to improve the handover process between the ICU and the general ward.

### Study design

To develop the implementation strategy, a complete and detailed plan to change the current way of working and improve quality, the Intervention Mapping (IM) method of Bartholomew will be used. IM is a systematic, iterative six-step process to develop an intervention program, based on theoretical, empirical and practical information [43]. Results of the previous phases (A to D) of this study will provide input for the IM method.

The first step is conducting a problem analysis to describe the healthcare problem, barriers and facilitators to change and the target population (*e.g.*, stage of behavioural change) (phase A, C, D). In step two, specific and feasible goals and change objectives (*e.g.*, what can be changed, what must be changed and who must make the change) are set. In step three, interventions from literature (phase B) are selected that correspond to the change objectives formulated in step two. Step four is the development of a tailored intervention program and pilot testing this program. In step five, the total implementation strategy is developed, including methods and tools for implementation. To evaluate the effects of the developed intervention program and implementation strategy, an effect and process evaluation is conducted in step six [43].

## Ethical approval

The study protocol has been presented to the Medical Ethical Committee of the Radboud University Nijmegen Medical Centre (registration number: 2011/460). They declared ethical approval was not required under Dutch National Law.

## Discussion

The goal of this study is to obtain more insight into current ICU discharge practices, and into the barriers and facilitators to the implementation of effective ICU discharge interventions. Analysis of the variation between ICU readmission rates and hospital mortality after ICU discharge reveals room for improvement. Improvement may be found in better adherence to effective ICU discharge interventions. In this study, a tailored implementation strategy will be developed based on theoretical and empirical information gathered. Insight into barriers and facilitators to the implementation of ICU discharge interventions is essential in deciding what kinds of activities should be developed to prevent suboptimal ICU discharges resulting in ICU readmissions and mortality. The knowledge obtained in this study should be used for the further implementation of ICU discharge interventions and can be used for implementation of handover interventions in other healthcare transition settings, such as operating theatre to ICU, operation theatre to general ward, hospital to hospital, or hospital to primary care interface.

This study uses a mixed methods approach, combining qualitative and quantitative research methods, to answer the research questions [42]. Therefore, a complete and in-depth view of the ICU discharge process is ensured, which is necessary for developing a tailored implementation strategy.

The definitions of the patients outcome measures in this study, ICU readmissions and hospital mortality after ICU discharge, are commonly used [16,37]. Therefore, our results can be compared to international literature. The NICE registry, a national database, contains data of almost every ICU patient in the Netherlands; 92% of all ICUs participate. This results in a nearly complete overview of characteristics of the ICU population and quality of ICU care. Also, the collection of data in the NICE database is standardized to ensure its quality.

In phase C, information about adherence is obtained by sending a questionnaire to all Dutch ICUs. A possible limitation of this method is response bias, which will be minimized by sending reminders by e-mail and follow-up calls. In addition, self-reporting adherence may result in overestimation. During the development of the questionnaire, questions that may invite socially desirable answers will be avoided.

The individual interviews and focus groups in phase D might raise questions about generalizability of the results. Therefore, the results of the interviews and focus groups will be quantified by a questionnaire sent to the entire Dutch intensivist population. The results of the questionnaire may also be subject to response bias, which also will be minimized by sending reminders.

The Intervention Mapping method used in phase E has not yet been proven more effective in comparison to other improvement methods. However, it is generally accepted that systematic development of tailored implementation strategies is preferred over intuitively selecting strategies [44].

Based on the results of this study, a tailor-made implementation strategy will be developed to improve the implementation of effective ICU discharge interventions in daily practice. In a subsequent study the cost effectiveness of the developed implementation strategy should be tested.

## Competing interests

The authors have no competing interests to declare.

## Authors’ contributions

MZ and HW contributed to the study design and coordination. NS en MZ drafted the manuscript. HW, JGH and GW revised the manuscript critically for important intellectual content. All authors read and approved the final manuscript.
